# The Pentameric Vertex Proteins Are Necessary for the Icosahedral Carboxysome Shell to Function as a CO_2_ Leakage Barrier

**DOI:** 10.1371/journal.pone.0007521

**Published:** 2009-10-21

**Authors:** Fei Cai, Balaraj B. Menon, Gordon C. Cannon, Kenneth J. Curry, Jessup M. Shively, Sabine Heinhorst

**Affiliations:** 1 Department of Chemistry and Biochemistry, The University of Southern Mississippi, Hattiesburg, Mississippi, United States of America; 2 Department of Biological Sciences, The University of Southern Mississippi, Hattiesburg, Mississippi, United States of America; 3 Department of Genetics and Biochemistry, Clemson University, Clemson, South Carolina, United States of America; University of Hyderabad, India

## Abstract

**Background:**

Carboxysomes are polyhedral protein microcompartments found in many autotrophic bacteria; they encapsulate the CO_2_ fixing enzyme, ribulose-1,5-bisphosphate carboxylase/oxygenase (RubisCO) within a thin protein shell and provide an environment that enhances the catalytic capabilities of the enzyme. Two types of shell protein constituents are common to carboxysomes and related microcompartments of heterotrophic bacteria, and the genes for these proteins are found in a large variety of bacteria.

**Methodology/Principal Findings:**

We have created a *Halothiobacillus neapolitanus* knockout mutant that does not produce the two paralogous CsoS4 proteins thought to occupy the vertices of the icosahedral carboxysomes and related microcompartments. Biochemical and ultrastructural analyses indicated that the mutant predominantly forms carboxysomes of normal appearance, in addition to some elongated microcompartments. Despite their normal shape, purified mutant carboxysomes are functionally impaired, although the activities of the encapsulated enzymes are not negatively affected.

**Conclusions/Significance:**

In the absence of the CsoS4 proteins the carboxysome shell loses its limited permeability to CO_2_ and is no longer able to provide the catalytic advantage RubisCO derives from microcompartmentalization. This study presents direct evidence that the diffusion barrier property of the carboxysome shell contributes significantly to the biological function of the carboxysome.

## Introduction

Carboxysomes, primitive organelles found in many autotrophic bacteria, consist of a thin polyhedral protein shell that surrounds a core of *r*ib*u*lose-1,5-*bis*phosphate *c*arboxylase/*o*xygenase (RubisCO) holoenzyme molecules (reviewed in [1], [2]). RubisCO catalyzes the first step of the inorganic carbon (C_i_) assimilation pathway, the Calvin-Benson-Bassham cycle. Sequestration of this rather inefficient enzyme into a protein microcompartment enhances RubisCO's catalytic performance and permits the bacteria that form carboxysomes to grow at appreciable rates at ambient CO_2_ concentrations. Mutants that are compromised in their ability to assemble functional carboxysomes have a *h*igh-*C*O_2_-*r*equiring (*hcr*) phenotype and do not grow at all or at greatly reduced rates, unless cultured in an atmosphere of elevated CO_2_. The molecular mechanism by which carboxysomes enhance the catalytic performance of RubisCO is in part attributable to the carbonic anhydrase activity of the shell-associated CsoSCA protein. This enzyme catalyzes the dehydration of cytosolic bicarbonate to CO_2_, the only C_i_ species RubisCO can use. In addition, the carboxysome shell constitutes a diffusional barrier for CO_2_, thereby preventing leakage of this C_i_ species out of the microcompartment and allowing the carboxysome to function as a CO_2_ trap that provides RubisCO in the interior with an on-demand supply of its substrate, CO_2_ [3].

Carboxysome-like polyhedral inclusions are not limited to autotrophic bacteria (reviewed in [4]). Microcompartments of similar appearance have also been observed in gram-negative and gram-positive heterotrophs during growth on certain carbon sources. Instead of RubisCO, these structures encapsulate enzymes that participate in the catabolism of the organic carbon species that induces microcompartment formation. The potential to form protein organelles is widespread among the bacteria [5], suggesting that microcompartmentalization of key metabolic steps constitutes a general regulatory strategy in prokaryotes.

All bacteria that have the potential to form carboxysome-like inclusions share two sets of genes that are thought to encode shell components common to all polyhedral protein microcompartments [2]. The genes for *b*acterial *m*icro*c*ompartment domain (BMC; pfam 00936) proteins are always present in multiple paralogs that encode highly abundant shell components. Representatives of α- and β-carboxysomes are the CsoS1 and CcmK/CcmO proteins, respectively. These proteins readily form homohexamers that, in turn, assemble into two-dimensional sheets in crystals [5], [6]. The BMC proteins are believed to form the facets of the icosahedral carboxysomes and related microcompartments [5], [6], [7], [8], [9], [10], [11].

The other protein family (pfam 03319) common to all polyhedral protein microcompartments is represented by the two paralogs *csoS4A* and *csoS4B* (formerly *orfA* and *orfB*, respectively) in the α-carboxysome operon, and by the *ccmL* gene in β-carboxysome gene clusters [12]. Recombinant CsoS4A from *H. neapolitanus* and CcmL from *Synechocystis* 6803 crystallize as pentamers, which are thought to occupy the vertices of the icosahedral carboxysome shell [10]. Since only 12 pentamers (or 60 monomers) of these proteins would be needed per carboxysome according to this model, it is not surprising that these proteins have so far eluded detection in purified carboxysome preparations. The elongated carboxysomes seen in a *ccmL* knockout mutant of *Synechococcus elongatus* PCC7942 (formerly *Synechococcus* sp. PCC7942, *Anacystis nidulans* R2) [13], [14] clearly indicate that the CcmL protein is important for β-carboxysome biogenesis, structure, and/or function. Since pentamers are required to close icosahedra built from hexamers, the phenotype of the mutant supports the proposed role of this protein in the carboxysome shell.

Although the current model of the carboxysome shell is able to assign functions to the CsoS1/CcmK and CsoS4/CcmL proteins, the structural roles of the remaining microcompartment shell proteins and of individual paralogs remain elusive. The structure of CsoSCA, the shell-associated carbonic anhydrase of α-carboxysomes encoded by *csoS3* [15] has been solved [16]. The enzyme is located on the inside of the shell [3]. Almost nothing is known about the CsoS2A and CsoS2B polypeptides, the two largest carboxysome components, regarding structure, location and function in the shell. Furthermore, the EutN protein of *Salmonella enterica*, which is the only CsoS4/CcmL homolog in that bacterium, crystallizes as hexamers and therefore begs the question whether another of the Eut proteins forms pentamers in the *e*thanolamine *ut*ilization (Eut) microcompartment [10].

To examine more closely the role of the pentamer-forming shell components, CsoS4A and CsoS4B, in the well characterized α-carboxysome of *Halothiobacillus neapolitanus*, a deletion mutant was created that is deficient in both paralogs. We have characterized the mutant with respect to growth phenotype and to carboxysome composition and function. Here we show that the CsoS4 proteins are apparently not essential determinants of carboxysome shape but are vital for the diffusion barrier properties of the shell.

## Results

### Wild type α-carboxysomes contain CsoS4A and CsoS4B polypeptides

Previous work had established that all genes of the *cso* operon, including *csoS4A* and *csoS4B*, are transcribed in *H. neapolitanus*. Furthermore, in cells grown in ambient CO_2_ the steady state transcript levels for those proteins that can be resolved by one-dimensional SDS-PAGE correlate with their abundance in the carboxysome [17]. These results suggested that all proteins encoded by the *cso* operon are present in α-carboxysomes. However, the low-abundance CsoS4 proteins were never reliably shown to be present in purified carboxysome preparations. Another factor that complicates the detection of these two putative carboxysome proteins is the inability to separate the paralogs CsoS1A and CsoS1C by conventional one-dimensional gel electrophoresis [1]. These two CsoS1 proteins have almost identical amino acid sequences and migrate as a single band that would probably also include the slightly smaller CsoS4A and CsoS4B polypeptides. To probe the presence of the low-abundance CsoS4 proteins, both polypeptides were overexpressed as histidine-tagged recombinant proteins in *E. coli* [10] and used to generate polyclonal antisera. As expected from the considerable similarity in primary sequence of both proteins [12], the antibodies raised against each CsoS4 protein cross-reacted equally well with the other paralog (not shown).

**Figure 1:**
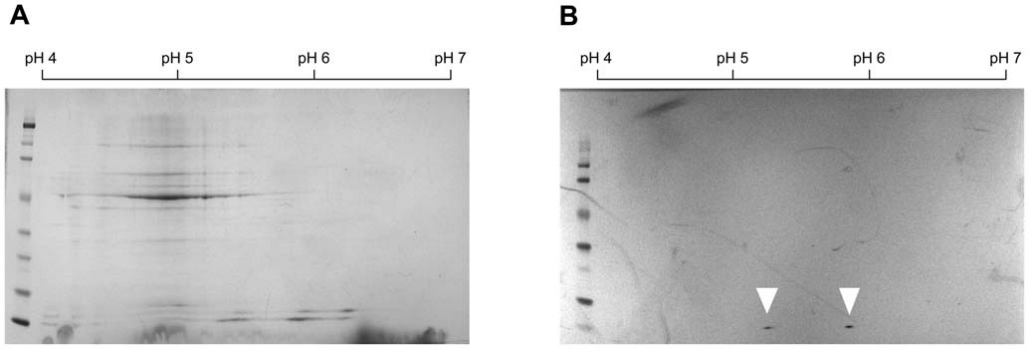
Two-dimensional separation of carboxysome shell proteins. Purified wild type carboxysomes were broken and a shell-enriched fraction was recovered after high-speed centrifugation [3]. (A) Proteins (90 µg) separated by two-dimensional SDS-PAGE and stained with Coomassie Blue. (B) A blot of the gel probed with anti-CsoS4B antiserum. The two immunoreactive spots representing CsoS4A (right) and CsoS4B (left) are indicated by white triangles.

To separate the CsoS4A and CsoS4B polypeptides based on differences in their predicted isoelectric points and distinguish them from the bands of the highly abundant CsoS1 proteins, a shell-enriched carboxysome fraction was subjected to two-dimensional gel electrophoresis. The pattern of stained polypeptide spots on the resulting gel was rather complex (Figure 1A) and illustrated the difficulty commonly encountered when attempting to completely dissociate the rather tight interactions of the carboxysome shell proteins. It was impossible to identify the low-abundance target CsoS4 polypeptide(s) among the stained spots. However, an imunoblot of the gel probed with anti-CsoS4B antiserum revealed two distinct polypeptide species of near-identical molecular mass but different isoelectric points (pIs) at the positions expected for CsoS4A (predicted pI  = 5.72) and CsoS4B (predicted pI  = 5.15), respectively (Figure 1B). These results indicated that the shell of *H. neapolitanus* wild type carboxysomes contains both CsoS4 paralogs. Furthermore, the intensity of both polypeptide spots was comparable on the immunoblot. Since the anti-CsoS4B antiserum crossreacts equally strongly with CsoS4A (not shown), it appears that both proteins are present in the carboxysome in similar copy numbers.

### The *HncsoS4AB::Km* mutant requires elevated CO_2_

**Figure 2:**
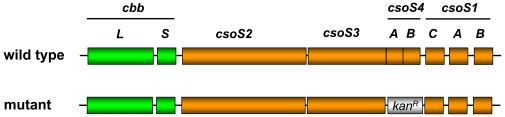
Genotype of *HncsoS4AB::Km*. The *csoS4A* and *csoS4B* genes of the *H. neapolitanus cso* operon were replaced with a *Kan^R^* cassette by homologous recombination *in vivo* as described previously [3], [18]. The mutant genotype was verified by genomic DNA sequencing. The wild type *cso* operon from the start codon of *cbbL* to the stop codon of *csoS1B* is 7686 nucleotides long.

Since CcmL, the only CsoS4 ortholog in *S. elongatus* PCC7942, is essential for β-carboxysome structure and function [13], [14], the role of the two paralogs CsoS4A and CsoS4B in α-carboxysomes was assessed by generating the *H. neapolitanus* double knockout mutant *HncsoS4AB::Km*. In this mutant, the coding sequences for the two CsoS4 proteins were replaced by a kanamycin resistance cassette (Figure 2) that carries its own promoter and therefore permitted expression of the downstream shell proteins CsoS1C, CsoS1A and CsoS1B.

**Figure 3:**
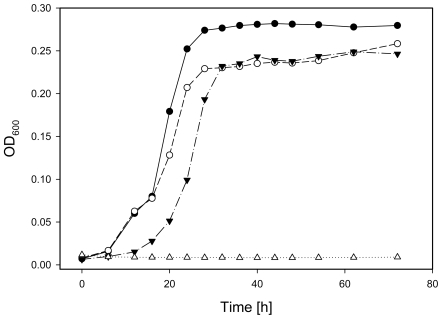
Growth curve of wild type and mutant *H. neapolitanus* cultures. Batch cultures were grown in air (wild type: open circles; mutant: open triangles) or in air supplemented with 5% CO_2_ (wild type: filled circles; mutant: filled triangles). The *HncsoS4AB::Km* mutant reaches lower cell densities than the wild type.

The *HncsoS4AB::Km* double knockout mutant displayed a strict *hcr* phenotype (Figure 3), showing no signs of growth for over 70 h in an ambient CO_2_ atmosphere. In air supplemented with 5% CO_2_, on the other hand, the mutant culture grew at a rate similar to that of the wild type but did not reach the same stationary phase cell density as the wild type. This phenotype suggested that deletion of both *csoS4* genes may have prevented the assembly of carboxysomes or that the function of mutant carboxysomes was severely impaired.

### The *HncsoS4AB::Km* mutant forms carboxysomes

**Figure 4:**
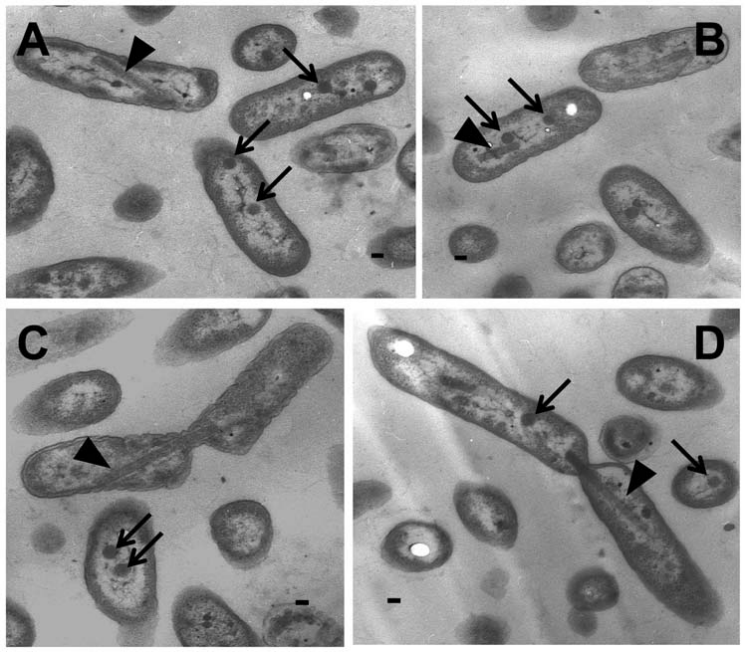
Transmission electron micrographs of *HncsoS4AB::Km* cells. The sectioned cells shown in (A) – (D) contain elongated carboxysomes (triangles) and carboxysomes of apparently icosahedral shape (arrows). Panels (C) and (D) show dividing cells with greatly elongated carboxysomes that extend across the constriction sites. Scale bar  = 100 nm.

Sectioned cells were examined by TEM to determine if the double knockout mutant produced carboxysomes. As seen in Figure 4A–D, many cells contained greatly elongated carboxysomes that were reminiscent of the malformed β-carboxysomes seen in the *S. elongatus* PCC7942 *ccmL* insertion mutant [13], [14]. Notably, several cells that were in the process of dividing contained very long mutant carboxysomes that extended across the constriction site between pairs of daughter cells and seemed to prevent complete fission (Figure 4C and D). The apparent interference of elongated carboxysomes with cell division could explain the inability of the *HncsoS4AB::Km* mutant culture to reach wild type cell densities but hardly accounted for the absolute *hcr* phenotype (Figure 3).

The observation that individual mutant cells contained carboxysomes of apparently normal appearance in addition to malformed ones (Figure 4A–D) was most surprising in light of the purported role of the CsoS4 proteins in the carboxysome [10]. In fact, “normal” carboxysomes seemed to outnumber elongated ones in cell thin sections. To obtain a more quantitative picture of the ratio of icosahedral to elongated carboxysomes in the mutant and in the wild type, carboxysomes were counted in a large number of cell thin sections in random fields of vision, regardless of the orientation in which cells and inclusions were cut (Table 1). In thin sections of wild type cells an average 0.58 carboxysome was visible per cell; no abnormally shaped microcompartments were observed in the 340 cells that were counted. Within the 376 mutant cell sections that were examined, 141 carboxysomes were observed, which translates to an average 0.38 carboxysome per mutant cell (approximately 2/3 of wild type). Less than 13% (18) of the mutant carboxysomes appeared to be elongated, suggesting that in *HncsoS4AB::Km* cells “normal” carboxysomes were an order of magnitude more prevalent than abnormally shaped ones.

**Table 1:** Number of Carboxysomes in *HncsoS4AB::Km* and Wild Type.

		Carboxysomes in Cell Thin Sections			Purified Carboxysomes	
	cells	total	elongated	per cell	total	elongated
**Mutant**	376	141	18	0.38	312	4
**Wild Type**	340	196	0	0.58	325	0

### Purified “normal” *HncsoS4AB::Km* carboxysomes outnumber elongated ones

**Figure 5:**
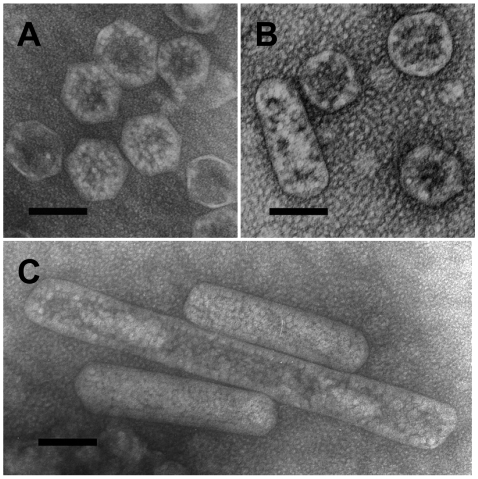
Transmission electron micrographs of purified *HncsoS4AB::Km* carboxysomes. The images in (A) – (C) show sucrose gradient-purified wild type (A) and mutant (B, C) carboxysomes that were negatively stained with ammonium molybdate. Scale bar  = 100 nm.

Cells from a chemostat culture of the *HncsoS4AB::Km* mutant were subjected to a standard carboxysome isolation procedure [3], [15], [18] because of concerns that the orientation in which the embedded mutant cells were sectioned might have influenced the apparent shape of the carboxysomes in cell thin sections and could have led to an over-estimation of “normal” carboxysomes. Following enrichment by differential centrifugation, carboxysomes were recovered from a band in the sucrose gradient that had formed at approximately the same position as one composed of wild type organelles. Transmission electron microscopy revealed that the majority of these carboxysomes were of normal appearance (Figure 5). Of 312 negatively stained carboxysomes counted in 20 randomly selected fields of vision, less than 2% had an abnormal shape; a comparable number of purified wild type carboxysomes contained no misshapen ones (Table 1).

### Mutant carboxysomes are devoid of CsoS4A and CsoS4B

**Figure 6:**
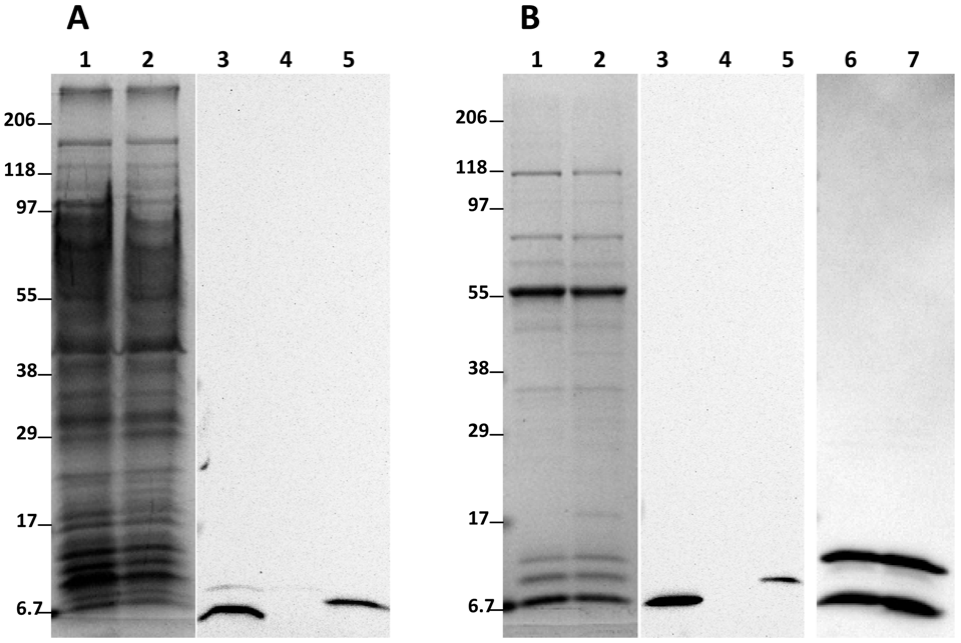
The *HncsoS4AB::Km* mutant does not produce CsoS4 protein. (A) Crude cell extract (50 µg) and (B) purified carboxysome (10 µg) proteins were separated by SDS-PAGE (lanes 1, 2). Blots were probed with anti-CsoS4B (lanes 3–5) and anti-CsoS1B (lanes 6, 7) antiserum. Wild type: lanes 1, 3, 6; mutant: lanes 2, 4, 7; lane 5: 10 ng rCsoS4 protein (1∶1 mixture of CsoS4A and CsoS4B). The reduced migration rate of the recombinant CsoS4 proteins is due to their C-terminal hexa-histidine tag. The mutant does not produce CsoS4A and CsoS4B.

Despite their low abundance in the carboxysome, the CsoS4 proteins could easily be visualized on an immunoblot of wild type cell extract probed with anti-CsoS4B antibody (Figure 6A, lane 3). By contrast, no CsoS4 protein was detectable in an equivalent amount of *HncsoS4AB::Km* mutant extract (Figure 6A, lane 4), indicating that the mutant cells, as expected, produced neither CsoS4A nor CsoS4B. The same result was obtained with immunoblots of purified carboxysomes (Figure 6B, lanes 3 and 4). As judged by the pattern of stained polypeptides on an SDS-polyacrylamide gel, the protein composition of mutant carboxysomes was essentially indistinguishable from that of their wild type counterparts (Fig. 6B, lanes 1 and 2). The immunoblot signals for the major shell components, the CsoS1 paralogs, were comparable in mutant and wild type particles (Figure 6B, lanes 6 and 7). These results suggested that mutant carboxysomes of normal shape could be formed in the absence of both CsoS4 paralogs.

### The shell of mutant carboxysomes is leaky

To determine whether a reduced carboxysomal CO_2_ fixation activity could explain the strict *hcr* phenotype of the *HncsoS4AB::Km* mutant, radiometric RubisCO activity assays were performed with sucrose gradient-purified mutant and wild type carboxysomes as described [3] (Table 2). Results from triplicate assays performed with two independent carboxysome preparations established that the V_max_ values for mutant carboxysomes (1.6±0.1 µmol·min^−1^·mg^−1^) were very close, if not identical, to those of wild type particles (1.8±0.1 µmol·min^−1^·mg^−1^). Surprisingly, the K_C_ for mutant carboxysomes (124±5.7 µM CO_2_) was lower by approximately 24% than that measured for their wild type counterparts (163.3±8.4 µM CO_2_). Since “normal” carboxysomes represented the majority of microcompartments purified from the mutant by a wide margin, these results suggested that, despite their apparently icosahedral shape and close-to-wild type protein composition, mutant carboxysomes lacking CsoS4A and CsoS4B were more permeable to C_i_ than wild type particles.

**Table 2:** CO_2_ Fixation Kinetics of *HncsoS4AB::Km* and Wild Type Carboxysomes.

	K_C_ a) (µM CO_2_)	Vmax (µmol·min^−1^·mg^−1^)
**Mutant**	124±5.7	1.6±0.1
**Wild Type**	163±8.4	1.8±0.1
**Wild Type** b)	177±16.3	1.7±0.1

a) K_C_ as defined in [3].

b) These values were taken from [3].

**Figure 7:**
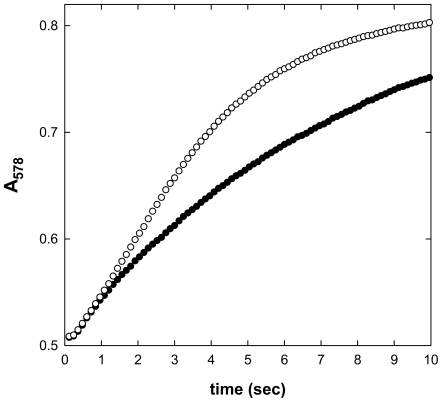
Catalytic activity of the carboxysomal carbonic anhydrase CsoSCA. The increase in pH upon dehydration of bicarbonate to CO_2_ by CsoSCA was followed using a colorimetric stopped-flow assay [19]. In the representative plot shown, the open circle trace shows the apparently faster kinetics (steeper initial slope) of CsoSCA activity in *HncsoS4AB::Km* mutant carboxysomes; the filled circle trace indicates wild type.

Since in wild type carboxysomes the activity of the shell-associated carbonic anhydrase, CsoSCA, and the limited permeability of the shell for inorganic carbon are crucial for the catalytic enhancement RubisCO derives from being compartmentalized [3], we measured the carbonic anhydrase activity of purified mutant and wild type carboxysomes with stopped-flow changing indicator assays [19] as described previously [20]. Clearly, the CsoSCA-catalyzed rate of bicarbonate dehydration was two to three fold faster in mutant carboxysomes than in wild type particles, as seen by the steeper slope of the pH change plots (Figure 7). The same difference in catalytic rates was also observed for the CO_2_ hydration reaction (not shown). Since the observed rate enhancement could not be explained by a significantly higher abundance of the carbonic anhydrase in mutant carboxysomes, this result also suggested that the shell of carboxysomes deficient in CsoS4A and CsoS4B is more permeable to inorganic carbon species.

## Discussion

Genes that are homologous to the carboxysomal *csoS4/ccmL* and *csoS1/ccmK* paralogs are found in the genomes of many bacteria [5], indicating that the genetic potential to compartmentalize key metabolic reactions in protein compartments is widespread among prokaryotes. The co-occurrence of the two types of genes that encode proteins believed to form facets (CsoS1/CcmK) and vertices (CsoS4/CcmL) of the icosahedral carboxysomes [10] further suggests that the polyhedral shells of all bacterial microcompartments share a common building principle, despite variations in shell protein structures [5], [6], [7], [8], [9], [10], [11], compositions and microcompartment functions [2], [4]. These two classes of shell proteins are clearly important for microcompartment structure and/or function.

CsoS4A and CsoS4B, the two smallest proteins encoded by the *cso* operon of *H. neapolitanus*, were shown to be present in α-carboxysomes in approximately equimolar, yet extremely low amounts. Their low abundance was expected based on the proposed function of CsoS4A pentamers at the carboxysome vertices and on their low steady state transcript levels [10], [17]. Likewise, the elongated carboxysomes that were apparent in thin sections of the *HncsoS4AB::Km* double knockout mutant were not surprising, since a similar phenotype had been observed in a *S. elongatus* PCC7942 *ccmL* mutant [13], [14], [21]. Interestingly, misshapen carboxysomes have occasionally also been observed in wild type cells of *H. neapolitanus* and other thiobacilli [22], [23] (Iancu et al., submitted), as well as in *S. elongatus* PCC7942 [24], where they are, however, rare compared to their abundance in the *H. neapolitanus csoS4AB:Km* double knockout and in the *S. elongatus ccmL* mutant. The existence of the occasional malformed carboxysome in wild type bacteria and the increased number of elongated carboxysomes in mutants deficient in the CsoS4/CcmL proteins suggested that the outcome of the carboxysome shell assembly process *in vivo* depends on the balanced availability of all structural components. Ectopic expression of the PduA protein in a *Salmonella enterica pduA* deletion mutant under conditions in which formation of the *p*ropane*d*iol *u*tilization (Pdu) microcompartment was induced resulted in a mixture of normal and abnormal microcompartments [25]. Likewise, when individual Pdu proteins were overexpressed in *E. coli* in addition to the entire *pdu* operon from *Citrobacter freundii*, inclusions of abnormal size and/or shape, indicative of non-physiological protein assemblies, were observed [26].

The coexistence of “normal” and elongated carboxysomes in mutant cells and among the population of purified mutant carboxysomes was unexpected and indicated that, contrary to the prediction of the current shell model [10], the two CsoS4 proteins are not essential shape determinants in the shell of α-carboxysomes. However, it is possible that subtle variations in shell structure and deviations from an icosahedral shape remained undetected in the transmission electron micrographs of negatively stained carboxysomes. To obtain a more comprehensive and physiologically relevant picture of the range of shapes exhibited by carboxysomes that lack the CsoS4 paralogs, a thorough cryo-electron tomography study would be required. This technique yields images of cells and organelles in their near-*in vivo* state and eliminates many of the artifacts that are often associated with the preparation of biological material for standard TEM.

The predominance of “normal” carboxysomes in the *HncsoS4AB::Km* mutant implicated other carboxysome shell component(s) as potential vertex proteins. The three CsoS1 paralogs come to mind since they are the major shell components of α-carboxysomes. However, since CsoS1A [6], and its homologs [5], [6], [7], [8], [10], [11] form crystals of hexamers or pseudo-hexamers, one would have to postulate that a BMC domain (pfam00936) protein is able to form pentamers as an alternative quaternary structure in the absence of both CsoS4 proteins. Precedents for multiple oligomeric states of proteins are found among those icosahedral viruses whose capsids are built from a single protein species (reviewed in [27]). Likewise, a presumed shell protein of β-carboxysomes, the CsoS1 ortholog CcmK2, also appears to assume quasi-equivalent oligomeric structures *in vitro* [5]. In light of this finding it is possible that one of the three CsoS1 paralogs of α-carboxysomes also possesses structural flexibility and fulfills the role of the missing CsoS4 proteins in the double knockout mutant *HncsoS4AB::Km*. It is noteworthy that the EutN protein crystallizes as hexamers [10]. Since this protein is the sole CsoS4 homolog in the *Salmonella enterica eut* operon, which encodes the *e*thanolamine *ut*ilization (Eut) microcompartment [28], pentameric assemblies would have been expected based on the similarity of its primary sequence to the CsoS4/CcmL proteins. Presumably, in the Eut microcompartment one of the other shell proteins, all of them CsoS1 homologs, may form pentamers.

The apparent structural flexibility of the two shell components common to bacterial microcompartments probably explains the considerable variability in size and shape of the organelles. While the exact composition of most microcompartments, including that of β-carboxysomes, is not known, the α-carboxysomes of *H. neapolitanus* are able to tolerate considerable deviations from their wild type protein composition without major effects on gross shell shape. For example, carboxysomes lacking the shell-associated carbonic anhydrase CsoSCA also appear to be of normal icosahedral shape and protein composition (*sans* CsoSCA) [3]. While the argument can be made that this lack of a detrimental effect on shell shape is easily explained by the low abundance of CsoSCA in the carboxysome [1], [29], Menon *et al.* clearly showed that the biogenesis of carboxysome shells does not require the presence of the highly abundant microcompartment cargo, RubisCO, and that carboxysomes can encapsulate chimeric and foreign RubisCO species [18].

The carboxysomes of the double knockout mutant *HncsoS4AB::Km*, despite their apparently normal shape, were clearly functionally impaired, as indicated by the absolute requirement of the mutant for elevated CO_2_. It was difficult to reconcile the strict *hcr* phenotype with the relatively small number of malformed carboxysomes present, particularly since a CsoSCA deletion mutant and several RubisCO replacement mutants [3], [18] are able to grow in air, albeit more slowly than the wild type. Cleary, the highly elongated mutant carboxysomes interfered with cell fission. However, since carboxysomes of apparently normal shape greatly outnumbered malformed organelles and not all cells contained elongated carboxysomes, this impairment alone should not result in an absolute *hcr* phenotype. A closer look at purified elongated carboxysomes revealed that, like carboxysomes of a RubisCO replacement mutant described previously [18], these microcompartments appeared to contain aggregated holoenzyme complexes (Figure 5B and C) of potentially compromised CO_2_ fixation ability; these may have contributed to, but do not explain entirely, the strict *hcr* phenotype of the mutant.

The lower value for K_C_ in mutant than in wild type carboxysomes was surprising in view of the normal appearance of the large majority of mutant carboxysomes and the low abundance of the CsoS4 proteins in wild type organelles. The value of K_C_ in the mutant was strikingly similar to that measured for broken wild type carboxysomes [3] and suggested that the permeability barrier function of the carboxysome shell for C_i_ is compromised in *HncsoS4AB::Km* organelles. Indeed, the apparently higher activity of the shell-associated carbonic anhydrase CsoSCA, which faces the inside of the carboxysome [3], suggested that this enzyme has more ready access to its substrate (CO_2_ or HCO_3_^−^) in mutant than in wild type carboxysomes. Shell “leakiness” for the RubisCO substrate CO_2_ is thereby implicated as the major factor leading to the strict *hcr* phenotype of the *HncsoS4AB::Km* mutant. Price *et al.* [13] measured the dissipation of the intracellular C_i_ pool following transfer of *S. elongatus* PCC4972 from light to dark and found that C_i_ was lost from the *ccmL* deletion mutant PVU at a considerably higher rate than from the wild type. The authors offered as two explanations for their observation: 1) the possibility of accelerated CO_2_ leakage from the mutant carboxysomes, and 2) the lower specific activity of the carboxysome-associated carbonic anhydrase. Our results support the first scenario. Apparently, carboxysomes lacking the shell proteins CsoS4A and CsoS4B, despite being able to assume an apparently normal shape and displaying near wild-type protein composition (*sans* CsoS4A and CsoS4B), lack the protein interactions that are necessary to form a “CO_2_-tight” shell. The mutant organelles therefore cannot provide the catalytic advantage RubisCO derives from being sequestered into wild type carboxysomes. The shell proteins CsoS4A and CsoS4B, although of very low abundance, clearly fulfill an important structural role in the shell and are vital to carboxysome function in the *c*arbon dioxide *c*oncentrating *m*echanism (CCM) of *H. neapolitanus*.

## Materials and Methods

### Bacterial strains and growth

*Halothiobacillus neapolitanus* c2 (ATCC23641) and the *HncsoS4AB::Km* mutant of this strain were grown at 30°C in a 2 L chemostat as described previously [3]. To establish a growth curve, 50 ml batch cultures of wild type and mutant were maintained in air and in air supplemented with 5% CO_2_. Growth was monitored by measuring the OD_600_ with a Beckman Coulter DU 800 spectrophotometer.

### Genomic DNA isolation

A 50 ml batch culture was grown to saturation and harvested by centrifugation at 9,000 rpm for 15 min at 4°C (Beckman JA 25.5 rotor). A Zymo Fungal/Bacterial DNA Kit (Zymo Research, Orange, CA) was used to purify genomic DNA, following the manufacturer's protocol with one modification: an additional 1 min spin at 10,000 rpm in a fresh 1.5 ml tube was performed immediately before elution of the DNA from the column.

### Recombinant CsoS4 protein expression and antiserum generation

The *csoS4A* (GenBank AAC32553.1) and *csoS4B* (GenBank AAC32554.1) expression clones in the pET-22b(+) vector (Novagen/EMD, Gibbstown, NJ) were generated by first amplifying the *csoS4A* and *csoS4B* coding sequences from pTn1 [30] using PfuUltra II DNA polymerase (Stratagene, La Jolla, CA) with primer pairs oAMscI/oArXhoIns and oBfNcoI/oBrXhoIns, respectively (Table S1). The primers contain restriction sites for in-frame ligation into the multi-cloning site of the vector. The resulting recombinant plasmids were transferred into chemically competent *E. coli* BL21(DE3) cells. Induction of protein expression and purification of the recombinant protein were as previously described [10]. Polyclonal antibodies were raised against both his-tagged CsoS4 proteins (Cocalico Biologicals, Reamstown, PA).

### Generation of the *HncsoS4AB::Km* double deletion mutant

The *HncsoS4AB::Km* deletion mutant was generated by replacing the *csoS4A* and *csoS4B* genes with a kanamycin resistance cassette in the *H. neapolitanus* genome. The kanamycin resistance cassette was PCR-amplified from the pCR-BluntII-TOPO vector (Invitrogen) with primers oAKmF and oBKmR (Table S1). The PCR product was electroporated into *E. coli* DY330 cells [31] containing the pTnE4.3 plasmid. This plasmid consists of a 4.3 kb EcoRI fragment from the *H. neapolitanus cso* operon (AF038430.1) that encompasses the region between the 3′-half of *csoS2* and the 3′-end of *csoS1B* in the vector pT7/T3α18 (J.M. Shively, unpublished). The *csoS4A*-*csoS4B* coding sequences in pTnE4.3 were replaced with the kanamycin resistance cassette *via* homologous recombination, yielding plasmid pTnE4.3-oABKm. This plasmid was introduced into exponentially growing *H. neapolitanus* cells by electroporation [32] to generate the *HncsoS4AB::Km* mutant by homologous recombination. Transformants were selected by growth on kanamycin-containing medium in a 5% CO_2_-enriched atmosphere. The mutant genotype was confirmed by diagnostic PCR amplification of genomic DNA with primer pairs S3f5368/1Cr6735 and orfAf6211/orfBr6472 (Table S1) and by sequence analysis (University of Maine DNA Sequencing Facility).

### Carboxysome purification and protein analyses

Carboxysome isolation and shell enrichment procedures were as previously described [3], [15], [18]. Proteins were separated by SDS-polyacrylamide gel electrophoresis (SDS-PAGE) in precast 10–20% gradient gels (Bio-Rad) and stained with Gelcode Blue (Thermo Scientific/Pierce). For two-dimensional electrophoresis, 88 µg of a carboxysome shell-enriched fraction were applied to an 11 cm, pH 4–7 ReadyStrip IPG strip (Bio-Rad). The strip was rehydrated and loaded with protein overnight at 50 V and 20°C. Isoelectric focusing proceeded at 20°C and 50 µA per strip at a rapid ramping rate to a maximum voltage of 8,000 V (total 40,000 V-h). Electrophoresis in the second dimension was as described for one-dimensional SDS-PAGE.

To detect the CsoS4 protein(s) in cell extracts and purified carboxysomes, gel blots were probed with rabbit polyclonal anti-CsoS4B antiserum as primary antibody and goat anti-rabbit horseradish peroxidase-conjugated IgG as secondary antibody (Santa Cruz Biotechnology Inc., Santa Cruz, CA). Blots were developed using SuperSignal West Pico chemiluminescent substrate (Thermo Scientific/Pierce) prior to visualizing immunoreactive bands with a VersaDoc imaging system (model 4000 MP; Bio-Rad).

Concentrations of purified proteins were determined with the BCA assay (Thermo Scientific/Pierce, Rockford, IL). Protein concentrations of cultured cells were estimated with a modified Lowry assay (Thermo Scientific/Pierce). Bovine serum albumin served as standard in both assays.

### Enzyme assays

RubisCO activity assays for the determination of K_C_ and V_max_ values of wild type and mutant carboxysomes were performed in triplicates with two independent carboxysome preparations as described previously [3].

To quantify the carbonic anhydrase activity of purified carboxysomes, colorimetric stopped-flow changing indicator assays [19] were performed to measure CO_2_ hydration and HCO_3_^−^ dehydration as described previously [3].

### Transmission electron microscopy

Exponentially growing wild type and mutant *H. neapolitanus* cells were gently centrifuged and the cell pellet resuspended in molten agarose (0.8% w/v in sterile water) at approximately 40°C. Bacteria in the solidified agarose plug were fixed for 2 h in 2.5% glutaraldehyde buffered in 0.1 M sodium cacodylate, pH 7.0. Cells were rinsed with 0.1 M sodium cacodylate, pH 7.0, and postfixed for 45 min in cacodylate-buffered 1% osmium tetroxide (pH 7.0). Cells were rinsed in distilled water, dehydrated in ethanol ranging from 50% to 100% followed by acetone, infiltrated in ERL 4206 resin (Spurr's), and cured at 70°C for 36 hours. Spurr's resin contained nonenyl succinic anhydride (26 g), vinyl cyclohexene dioxide (10 g), diglycidyl ether (6 g), and dimethylaminoethanol (0.2 g). Ultrathin sections were taken using a Porter-Blume MT-2B ultramicrotome with a diamond knife and collected on 200 mesh copper grids. Grids were stained in a moist chamber on drops of lead citrate [33] for 15 min, followed by 2% aqueous uranyl acetate for 15 min. Purified carboxysomes were negatively stained as previously described [3]. Specimen were viewed using a Zeiss 109T transmission electron microscope.

## Supporting Information

Table S1 PCR primers used in this study.
(0.04 MB DOC)
