# Supplementary material for: The Pentameric Vertex Proteins Are Necessary for the Icosahedral Carboxysome Shell to Function as a CO2 Leakage Barrier
Source: PLoS One. 2009 Oct 21;4(10):e7521. doi: 10.1371/journal.pone.0007521 (PMC2760150; doi:10.1371/journal.pone.0007521)
Supplement: Table S1 — PCR primers used in this study. (0.04 MB DOC) [file pone.0007521.s001.doc]

**Supplementary Table S1. Primers Used in this Study.**

| **Target Gene(s)** | **Primer** | **Primer Sequence** |
| --- | --- | --- |
| **Recombinant CsoS4 protein expression** | | |
| *csoS4A* | oAfMscI | CGATGGCC**ATGAAAATCATGC** |
|  | oArXhoIns | GGTCCTCGAG**CTCACCATTCC** |
| *csoS4B* | oBfNcoI | CGACC**ATGGAAGTAATGCGCG** |
|  | oBrXhoIns | GGTCCTCGAG**AGTTACCCAGTGATCG** |
| **Generation of the *kan^R^* insertion into *csoS4AB*** | | |
| *csoS4A-csoS4B* | oAKmF | **GGCGCAGGATATCGGAAGCCCGATTGAAGAGGTTGCATCCGCATGA***CCGGAATTGCCAGCTGGG* |
|  | oBKmR | **CTCTCCTCAATAAAGACTCATTCAAAATCAACTCATCTAGCGATGG***TCAGAAGAACTCGTCAAGAAGGCG* |
| **Diagnostic PCR for *HncsoS4AB::Km* mutant** | | |
| *csoS3-csoS1C* | S3f5368 | **CACCTCTGACCCGACACACTCTG** |
|  | 1Cr6735 | **CTGCTGCCATTTCTTCTCTCC** |
| *csoS4A-csoS4B* | orfAf6211 | **TCAACAAACCGTATTGCTGATATG** |
|  | orfBr6472 | **CTGCGTGTTGCGATTAGG** |

The numbers in the primer names refer to the position of the 5'-end with respect to the numbering system used in NCBI records AF038430; the letters f and r indicate forward and reverse primer, respectively. Underlined regions mark restriction sites; bold letters indicate regions that are homologous to *H. neapolitanus* genomic DNA; italic letters mark homology to the *Kan*^r^ cassette. All oligonucleotides were synthesized by Integrated DNA Technologies (Coralville, IA).
